# VisualZoneR: A computational protocol to identify compartmental zones from single-cell spatial transcriptomics using R

**DOI:** 10.1016/j.xpro.2024.103196

**Published:** 2024-07-26

**Authors:** Thomas Kerzel, Stefano Beretta, Luigi Naldini, Mario Leonardo Squadrito

**Affiliations:** 1 San Raffaele Telethon Institute for Gene Therapy, IRCCS San Raffaele Scientific Institute, 20132 Milan, Italy; 2 Vita Salute San Raffaele University, 20132 Milan, Italy

**Keywords:** Bioinformatics, Single Cell, Cancer, Gene Expression

## Abstract

VisualZoneR is an R-based technique used to analyze spatial transcriptomics data generated by employing Visium or Visium HD technology. Here, we present a protocol to identify compartmental zones from single-cell spatial transcriptomics using VisualZoneR. We describe steps for identifying distinct zones ranging from healthy liver tissue to inner metastatic areas and measuring transcriptomic changes. We then detail procedures for integrating distinct samples and grouping transcriptomic spots into compartmental zones according to their relative distance from the tumor/liver parenchyma boundary.

## Highlights

• VisualZoneR is an R-based technique to analyze spatial transcriptomics data
• Steps for identifying distinct zones according to the distance to a defined boundary
• Guidance on grouping of transcriptomic spots into compartmental zones

## Abstract (editor highlights)

Publisher’s note: Undertaking any experimental protocol requires adherence to local institutional guidelines for laboratory safety and ethics.

## Graphical abstract

**Figure:**
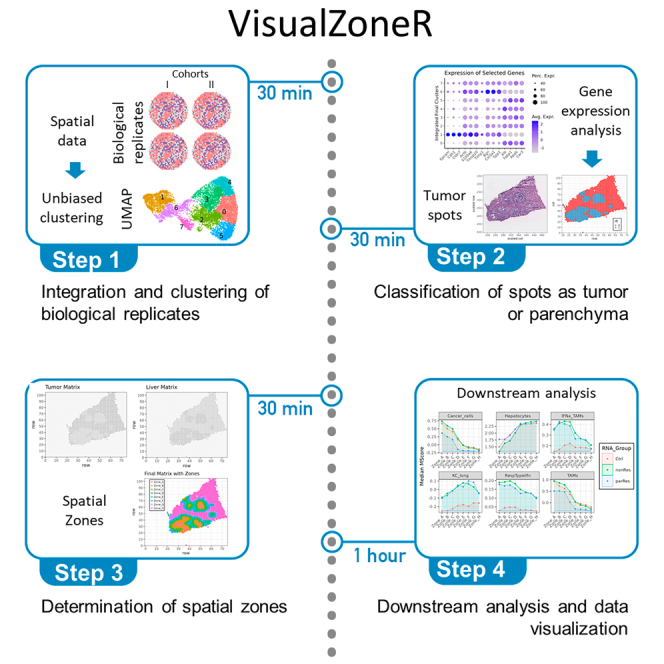

## Before you begin

### Overview

In depth understanding of the tumor microenvironment (TME) is of pivotal importance for the development of new cancer therapies.^1^ Of note, the interface between the tumor and the healthy tissue is recognized as the most immune active area, while the center of the tumor exhibits immunosuppressive features.^2^^,^^3^ It has been shown that the spatial organization of diverse immune cells can predict clinical outcome, thereby influencing the selection of treatment strategies. Therefore, spatially resolved transcriptomic analysis, such as Visium technology from 10X Genomics, offers a powerful tool for a more in-depth characterization of the TME and was rightfully awarded the Method of the Year in 2020.^4^ Nevertheless, the biggest challenges for the analysis of spatial transcriptomics remain the unbiased integration of biological replicates and the interpretation of the data. VisualZoneR is a computational approach developed in R for the integration and analysis of spatial transcriptomics data generated using Visium technology. In the case study presented here, we applied VisualZoneR to mouse liver sections containing liver metastases, with a focus on analyzing transcriptomic changes occurring in the transition from healthy tissue to inner metastatic areas. VisualZoneR enables grouping of transcriptomic spots into distinct compartmental zones according to their relative distance from the metastasis/liver parenchyma boundary. Moreover, VisualZoneR enables the unbiased integration of biological replicates and comparison between different cohorts as described by our group in a previous study.^5^ VisualZoneR can be used for analyzing various types of tumors and other studies where spatial transcriptomics is employed to examine transitions between different tissues. In addition to that, VisualZoneR can also be employed to analyze datasets generated using Visium HD technology (from 10X Genomics), a high-resolution spatial transcriptomics technology enabling spatial gene expression analysis at single cell scale.

### Description of spatial transcriptome dataset

We generated the spatial transcriptomic dataset by using Visium technology from 10X Genomics, a spatial transcriptomics technology with 6.5 × 6.5 mm capture areas covered with about 5,000 spots of distinct oligonucleotides. The distance between gene expression spots are about 100 μm. Visium technology was applied according to manufacturer’s instruction using the manuals “Methanol Fixation, H&E Staining & Imaging for Visium Spatial Protocols (10x Genomics / CG000160 Rev D)” (https://www.10xgenomics.com/support/spatial-gene-expression-fresh-frozen/documentation/steps/tissue-staining) using an Aperio ePathology digital scanner (Leica Biosystems) for image acquisition, and “Spatial Gene Expression Reagent Kits (10X Genomics / CG000239 Rev F)” (https://www.10xgenomics.com/support/spatial-gene-expression-fresh-frozen/documentation/steps/library-construction/visium-spatial-gene-expression-reagent-kits-user-guide) as described by our group in a previous study.^5^ The optimal permeabilization time may vary for different tissue types, and thus, we initially investigated this factor using 7, 14, 21 or 28 min. For this study, a permeabilization time of 14 min was used. Sequencing was performed using the Illumina NovaSeq 6000 platform according to the manual. The demultiplexing, barcoded processing, gene counting and aggregation were performed by using the Space Ranger software v1.2.2 (https://support.10xgenomics.com/spatial-gene-expression/software/pipelines/latest/what-is-space-ranger) and employing the mouse reference genome assembly mm10. The dataset can be downloaded at: https://www.ncbi.nlm.nih.gov/geo/query/acc.cgi?acc=GSE221359.

### Download R, RStudio, and required R packages

**Timing: ∼2 h depending on the computational power**

1. The open-source software R provides a platform commonly used for statistical analysis and graphical data representation. To download R, refer to https://www.r-project.org/. We executed VisualZoneR using R version 4.0.3.
2. Download RStudio, an integrated development environment, from the following link: https://www.rstudio.com/products/rstudio/. We run VisualZoneR on RStudio version 1.3.1093.

install.packages("remotes")

remotes::install_version("Seurat", "4.1.1", repos = c("https://satijalab.r-universe.dev", getOption("repos")))

install.packages("ggplot2")

devtools::install_github("thomasp85/patchwork")

install.packages("dplyr")

install.packages("cowplot")

BiocManager::install("OLIN")

BiocManager::install("fgsea")

install.packages('gplots')

install.packages("openxlsx")

install.packages("stringr")

install.packages("reshape2")

***Note:*** R packages are collections of functions and compiled code that extend R functionality. To run VisualZoneR, the following packages are necessary: Seurat, ggplot2, patchwork, dplyr, cowplot, OLIN, fgsea, gplots, openxlsx, stringr and reshape2. The versions of the packages that we employed are indicated in the key resources table.

**CRITICAL:** Other versions of the packages might result in unforeseen error messages. Note that this can be avoided employing the indicated version of R and packages. This code is mostly based on Seurat; therefore, other versions of Seurat could compromise the functionality code. The Matrix package version should be 1.6–4 or lower.

## Key resources table

**Table:** 

REAGENT or RESOURCE	SOURCE	IDENTIFIER
**Deposited data**		

Spatial transcriptomic data from murine liver metastasis	DOI: https://doi.org/10.1016/j.ccell.2023.09.014	GEO: GSE221359
R scripts and supplementary files	DOI: https://doi.org/10.1016/j.ccell.2023.09.014	http://www.bioinfotiget.it/gitlab/custom/squadrito_livertumor2022/squadrito_livertumor2022_spatial

**Software and algorithms**		

R 4.0.3	http://www.R-project.org/	N/A
Seurat package (v4.1.1)	https://satijalab.org/seurat/	N/A
ggplot2 package (v3.4.4)	https://ggplot2.tidyverse.org/	N/A
patchwork package (v1.2.0)	https://patchwork.data-imaginist.com/	N/A
dplyr package (v1.1.4)	https://dplyr.tidyverse.org/	N/A
cowplot package (v1.1.2)	https://wilkelab.org/cowplot/	N/A
OLIN package (v1.80.0)	https://www.bioconductor.org/packages/release/bioc/html/OLIN.html	N/A
fgsea package (v1.28.0)	https://bioconductor.org/packages/release/bioc/html/fgsea.html	N/A
gplots package (v3.1.3)	https://github.com/talgalili/gplots	N/A
openxlsx package (v4.2.5.2)	https://ycphs.github.io/openxlsx/index.html	N/A
stringr package (v1.5.1)	https://stringr.tidyverse.org/	N/A
reshape2 package (v1.4.4)	https://cran.r-project.org/web/packages/reshape2/index.html	N/A

## Step-by-step method details

### Download the dataset, perform quality control, and integrate samples

**Timing: few minutes per sample, depending on the computational power**

In the first step of VisualZoneR, the dataset is downloaded (in case the exemplary dataset described here is used) and provided in the correct format and data location. Furthermore, the quality of the individual transcriptomic spots is assessed in terms of number of RNAs detected ('nCount_Spatial'), number of genes detected ('nFeature_Spatial'), percentage of mitochondrial genes ('percent_mito') and percentage of hemoglobin genes ('percent_hb'). Subsequently, the dataset is filtered by setting the threshold for the number of detected genes ('nFeature_RNA') to equal or higher than 500 and the percentage of mitochondrial genes ('percent_mito') to equal or lower than 20%. Furthermore, this step describes the preparation of a single object in which the spatial transcriptomic spots of all samples are integrated and normalized.

1. Download the dataset and prepare the input data.
  a. Download data from https://www.ncbi.nlm.nih.gov/geo/query/acc.cgi?acc=GSE221359 and extract the files.
  b. Prepare the files as follows:
    i. The folders containing the images of each sample retrieved from the '<sample>_spatial.tar.gz' file are placed in the data input folder (hereon 'geo_dir') and named by concatenating the sample name and the suffix (e.g., Sample3_spatial).
    ii. The '.h5′ files, for each sample, are placed in the 'geo_dir' folder and named by concatenating the sample name and the suffix '_filtered_feature_bc_matrix.h5' (e.g., Sample3_filtered_feature_bc_matrix.h5).

***Note:*** This step applies only if the exemplary dataset is used. Alternatively, the dataset of interest which should be analyzed using VisualZoneR is provided as input data.

**Figure 1:**
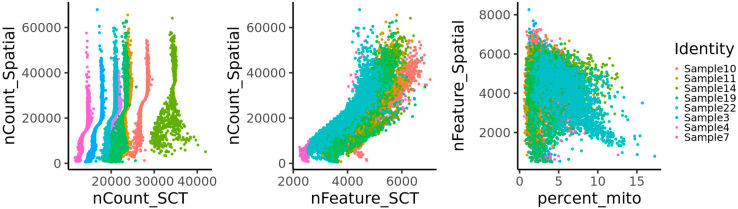
Quality control plots Scatterplots showing the relations among the number of features (nFeature_Spatial), counts (nCount_Spatial), and percentage of mitochondrial genes (percent_mito), colored by individual samples.
  c. Process the integrated object by computing PCA, UMAP embeddings, and clusters.
    ***Note:*** The number of PCs computed, the number of those used in the UMAP and the clustering resolutions strictly depends on the dataset. One possibility to determine the number of PCs used in the 'RunUMAP' function is based on the elbow plot in which principal components are ranked based on the percentage of variance explained by each one. By selecting a number of PCs in correspondence to the 'elbow', it is suggested that the majority of the true signal is captured. The parameter for resolution in the 'FindClusters' function determines the granularity of the clustering. The parameter should be adjusted to retrieve a meaningful clustering. As in this case where two tissues should be distinguished, a low level of granularity is sufficient. Further indications on how to choose these parameters can be found at the following webpage:
    https://satijalab.org/seurat/articles/pbmc3k_tutorial.html.
  d. Merge cluster that are redundant or poorly represented and save the Seurat object.
    ***Note:*** Using different versions of R or R packages may cause differences in the clustering and alter the requirements for merging clusters.
    #4c:
    crc.integrated <- ScaleData(crc.integrated, verbose = FALSE)
    crc.integrated <- RunPCA(crc.integrated, npcs = 70, verbose = FALSE)
    ElbowPlot(crc.integrated, ndims = 70)
    DefaultAssay(crc.integrated) <- "integrated"
    # UMAP and Clustering
    crc.integrated <- RunUMAP(crc.integrated, dims = 1:25, seed.use = 123)
    crc.integrated <- FindNeighbors(crc.integrated, reduction = "umap", dims = 1:2, force.recalc = T)
    crc.integrated <- FindClusters(crc.integrated, resolution = 0.1)
    Idents(crc.integrated) <- "integrated_snn_res.0.1"
    crc.integrated <- SCTransform(crc.integrated, assay = "Spatial", new.assay.name = "SCTintegrated")
    #4d:
    crc.integrated@meta.data$integrated_FinalClusters <- crc.integrated@meta.data$integrated_snn_res.0.1
    crc.integrated@meta.data$integrated_FinalClusters[crc.integrated@meta.data$integrated_FinalClusters == 8] <- 2
    png(filename = paste(out_dir, "DimPlot_integrated_refined.png", sep = "/"), width = 1200, height = 1000, res = 100)
    print(DimPlot(crc.integrated, reduction = "umap", group.by = "integrated_FinalClusters", label = T, pt.size = 1.5))
    dev.off()
    saveRDS(crc.integrated, file = paste(out_dir, "crc.integrated.rds", sep = "/"))
    Here, we provide an example of visualization of the computed clusters in the integrated object (Figure 2).
    # Figure 2: Integrated Final Clusters
    df <- FetchData(object = crc.integrated, c("UMAP_1", "UMAP_2", "integrated_FinalClusters"))
    p <- ggplot(data = df, mapping = aes(x = UMAP_1, y = UMAP_2, color = integrated_FinalClusters)) + theme_void() + theme(legend.position = "none") + ggtitle("Integrated Final Clusters") + geom_point(size = .25) + scale_color_hue()
    p <- LabelClusters(plot = p, id = "integrated_FinalClusters", size = 5, color = "black")
    ggsave(filename = paste(out_dir, "DimPlot_integrated_refined.png", sep = "/"), plot = p, width = 8, height = 6, units = "cm", dpi = 300)

2. Load required R packages, setting of directories and define the quality control parameters.
  a. Load the required R packages.
  b. Set input and output directories.
    ***Note:*** Replace 'geo_dir' with the path of the folder where the input data are stored. Additionally, set the output path 'wdir' as needed.
  c. Define the quality control parameters. Mitochondrial genes contain the prefix 'ˆmt-'
    ***Note:*** thresholds can be defined by the user according to quality requirements and may differ dependent on the sample or experiment.
    #2a:
    library(Seurat)
    library(ggplot2)
    library(patchwork)
    library(dplyr)
    library(cowplot)
    library(OLIN)
    library(fgsea)
    library(gplots)
    library(stringr)
    library(openxlsx)
    #2b:
    wdir <- "∼/squadrito_livertumor2022_spatial" #replace by required working directory
    geo_dir <- "∼/GEO_data" #use path where you placed data files
    data_dir <- paste(wdir, "data", sep = "/")
    out_dir <- paste(wdir, "results", sep = "/")
    dir.create(path = data_dir, showWarnings = F)
    dir.create(path = out_dir, showWarnings = F)
    #2c:
    analysis_params <- list(
     min.feature = 500, # min nFeature_RNA
     max.pc.mito = 20, # max percent.mt
     mito.prefix = "ˆmt-" # mito prefix
    )
3. In this section of VisualZoneR, a loop is employed to load all samples as individual objects, filter the transcriptomic spots by the previously set quality control parameters, visualize all assessed quality parameters, annotate each sample according to its treatment cohort as Responder (“parRes”), Resistant (“nonRes”) and Control (“Ctrl”), prepare the objects for integration, and generate a list containing all objects.
  a. Load individual images.
    ***Note:*** The folder containing the images of each sample retrieved from the '<sample>_spatial.tar.gz' file has to be placed in the 'geo_dir' directory and named by fusing the sample name and the suffix '_spatial' (*e.g.* Sample3_spatial). Similarly, the '.h5′ file for each sample has to be placed in the 'geo_dir' folder and named by fusing the GSM number, sample name and the suffix '_filtered_feature_bc_matrix.h5' (*e.g.* GSM6859066_Sample3_filtered_feature_bc_matrix.h5). If a dataset other than the exemplary dataset described here should be analyzed, the input information such as file names and cohort description ('treatmentGroups', provided in the order of samples loaded) should be adjusted accordingly.
  b. Annotate the quality control features and filter out spatial transcriptomic spots that do not meet the required parameters.
  c. Perform SCTransformation and normalization on each individual sample.
  d. Add sample information and prepare for integration by normalizing and finding variable features and generate a list containing all objects.
    #3a:
    samples <- c("Sample3", "Sample4", "Sample7", "Sample10", "Sample11", "Sample14", "Sample19", "Sample22")
    GSM_Numb <- c("GSM6859066_", "GSM6859067_", "GSM6859068_", "GSM6859069_", "GSM6859070_", "GSM6859071_", "GSM6859072_", "GSM6859073_")
    treatmentGroups <- c("Ctrl", "nonRes", "Ctrl", "parRes", "parRes", "nonRes", "Ctrl", "parRes")
    reference.list <- c()
    for (i in 1:length(samples)) {
     sample <- samples[i]
     GSM <- GSM_Numb[i]
     obj_img <- Read10X_Image(image.dir = paste(geo_dir, paste0(sample, "_spatial"), sep = "/"))
     obj <- Load10X_Spatial(data.dir = geo_dir, filename = paste0(GSM,sample, "_filtered_feature_bc_matrix.h5"), image = obj_img, slice = paste0(sample, "img"))
    #3b:
     obj <- PercentageFeatureSet(obj, analysis_params$mito.prefix, col.name = "percent_mito")
     obj <- PercentageFeatureSet(obj, "ˆHb.∗-", col.name = "percent_hb")
     obj <- obj[, obj$nFeature_Spatial > analysis_params$min.feature & obj$percent_mito < analysis_params$max.pc.mito]
    #3c:
     obj <- SCTransform(object = obj, assay = "Spatial", return.only.var.genes = FALSE, verbose = FALSE)
     obj <- NormalizeData(obj, verbose = FALSE, assay = "Spatial")
     obj <- GroupCorrelation(object = obj, group.assay = "Spatial", assay = "Spatial", slot = "data", do.plot = FALSE)
     obj <- GroupCorrelation(object = obj, group.assay = "Spatial", assay = "SCT", slot = "scale.data", do.plot = FALSE)
    #3d:
     obj@meta.data$orig.ident <- sample
     obj@meta.data$TreatGroups <- treatmentGroups[i]
     DefaultAssay(obj) <- "SCT"
     obj <- NormalizeData(obj, verbose = FALSE)
     obj <- FindVariableFeatures(obj, selection.method = "vst", nfeatures = 500, verbose = FALSE)
     reference.list <- c(reference.list, obj)
    }
4. Integrate all samples into a single object and process it.
  a. Integrate samples into one object.
    ***Note:*** The number of dimensions to use depends on the specific dataset.
  b. Assess quality control parameters for the integrated object (Figure 1).
    ***Note:*** Important parameters are “nFeature_Spatial”, which represents the number of genes detected in each cell, and “nCount_Spatial”, which is the total number of molecules detected in a cell. In a similar way “nFeature_SCT” and “nCount_SCT” represent genes and molecules respectively but after the SCTransformation. Moreover, “percent_mito” corresponds to the percentage of mitochondrial genes in each cell. An important aspect, when dealing with multiple samples together, is to verify that all the samples behave in the same way, to obtain a good integration.
    #4a:
    crc.anchors <- FindIntegrationAnchors(object.list = reference.list, dims = 1:30)
    crc.integrated <- IntegrateData(anchorset = crc.anchors, dims = 1:30)
    #4b:
    DefaultAssay(crc.integrated) <- "integrated"
    plot1 <- FeatureScatter(crc.integrated, feature1 = "nCount_SCT", feature2 = "nCount_Spatial", group.by = "orig.ident", plot.cor = F)
    plot2 <- FeatureScatter(crc.integrated, feature1 = "nFeature_SCT", feature2 = "nCount_Spatial", group.by = "orig.ident", plot.cor = F)
    plot3 <- FeatureScatter(crc.integrated, feature1 = "percent_mito", feature2 = "nFeature_Spatial", group.by = "orig.ident", plot.cor = F)
    png(filename = paste(out_dir, "IntegratedObj_QC.png", sep = "/"), width = 1200, height = 1000, res = 100)
    print(plot1 + plot2 + plot3)
    dev.off()

**Figure 2:**
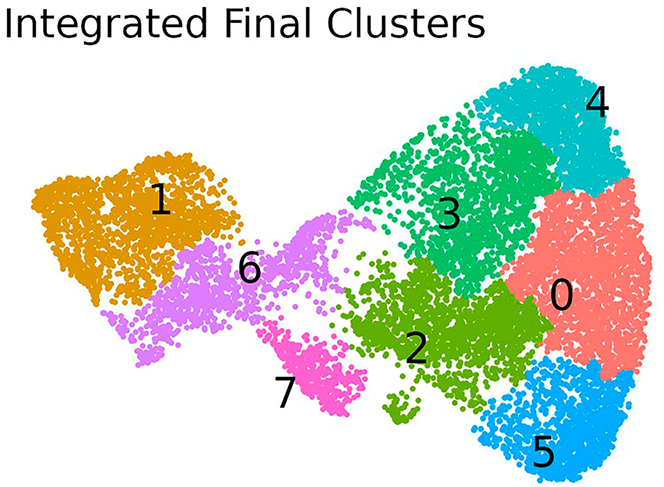
Clustering of the integrated object UMAP plot showing the clusters computed on the integrated object.

### Grouping of spatial spots into compartmental zones according to their relative distance from the metastasis/liver parenchyma boundary

**Timing: few minutes**

In this step of VisualZoneR, we evaluate the relative distance of each spot from the metastasis/liver parenchyma boundary using a moving average function. Subsequently, we categorize the spatial spots into 8 distinct compartmental zones, extending from the center of the tumor to the tumor-distant liver parenchyma.

***Note:*** Transcriptomic spots from clusters 1 and 6 were identified as metastatic areas while all the other clusters represent hepatic parenchyma. However, for other datasets or other types or analyses the user may select other clusters to define the tissue/zone of interest.

**Figure 3:**
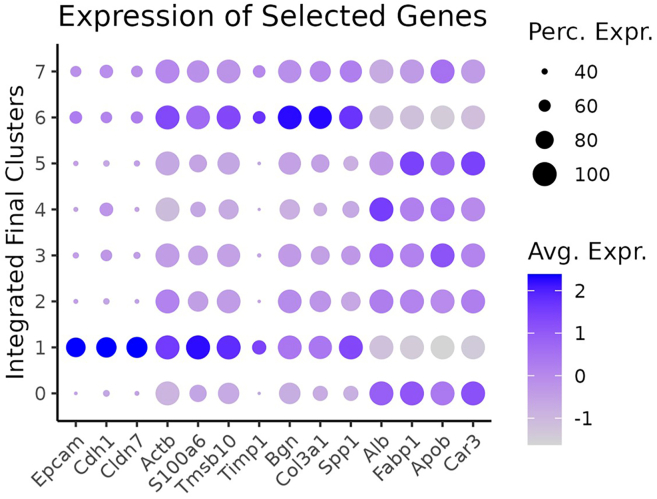
Expression of selected genes Dotplot showing the expression of metastasis-related and liver-related genes in the computed clusters, colored by their average expression. Size represents the percentage of cells expressing the corresponding gene in the specific cluster.

***Note:*** The list of genes used for classification of the sports can be selected according to the users' preferences and experimental goal. Indeed, other genes could be selected to enable precise cell distinction for other tissues or tumor models.

5. Classification of spatial spots as metastatic or liver parenchyma based on the previously computed clustering and by assessing the expression of common colorectal cancer-related genes (*Epcam*, *Cdh1*, *Cldn7*, *Actb*, *S100a6*, *Tmsb10*, *Timp1*, *Bgn*, *Col3a1*, *Saa3*, *Spp1*) and liver-related genes (*Alb*, *Fabp1*, *Apob*, *Car3*) in each cluster (Figure 3).

# Figure 3: Expression of Selected Genes

p <- DotPlot(crc.integrated, features = c("Epcam", "Cdh1″, "Cldn7″, "Actb", "S100a6", "Tmsb10", "Timp1", "Bgn", "Col3a1", "Spp1", "Alb", "Fabp1", "Apob", "Car3"), group.by = "integrated_FinalClusters", dot.scale = 4) + theme_classic(base_size = 10) + theme(axis.text.x = element_text(angle = 45, hjust = 1), legend.key.size = unit(.5, "cm")) + ggtitle("Expression of Selected Genes") + guides(color = guide_colorbar(title = "Avg. Expr."), size = guide_legend(title = 'Perc. Expr.')) + xlab("") + ylab("Integrated Final Clusters")

ggsave(filename = paste(out_dir, "DotPlot_Genesignature_Cluster.png", sep = "/"), plot = p, width = 10, height = 8, units = "cm", dpi = 300)

**Figure 4:**
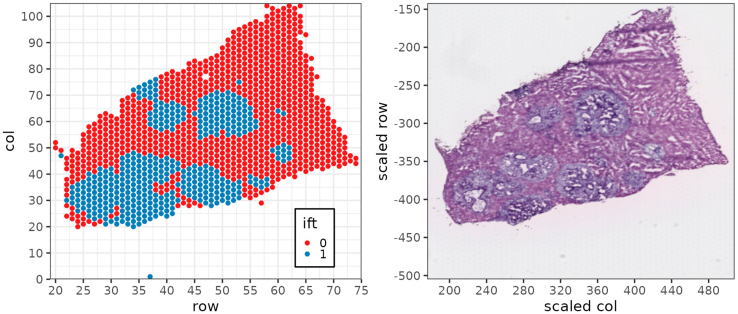
Spatial representation of the “ift” values of the transcriptomic spots and H&E image of sample 4 Scatter plot of a sample showing the cells of the matrix colored based on the selected cluster of interest (left) and the corresponding H&E image (right).
  c. Create a binary matrix, 'msub1' in which all coordinates of spots covering a metastatic area are annotated as 1, while spots covering a liver parenchyma area are annotated as 0 (Figure 5).

6. Loop in which VisualZoneR groups the spatial spots for each sample individually into compartmental zones according to their relative distance from the metastasis/liver parenchyma boundary and annotates them accordingly.
  a. Create a subset object for each sample and extract the coordinates for each spatial spot.
  b. Annotate spatial spots belonging to clusters classified as metastatic.
    ***Note:*** If clusters identified as metastatic (here cluster 1 and 6) differ due to changes in the clustering, spots of clusters annotated as 1 in the Coord$ift slot (identified by 'Coord$integrated_FinalClusters == 1 | Coord$integrated_FinalClusters == 6′) must be adjusted. For other applications beyond the analysis of liver metastasis, clusters summarizing spots covering the tissue category of interest must be annotated as 1 accordingly.
    We provide an example of visualization of the “ift” values, which stands for “*if tumor”* and identifies spots as liver parenchyma (“ift” = 0) or metastasis (“ift” = 1) in the matrix of sample 4 with the corresponding H&E image. There is precise overlap between spots identified as metastatic area (“ift” = 1, Figure 4, **left panel**) and the metastasis that can be observed in the H&E staining (Figure 4, **right panel**). Of note, we observed similar results for all 8 sections analyzed in this study. The strong overlap confirms that this method enables an automated distinction between spots covering metastatic and non-metastatic hepatic regions, which is key for further classification of spots into distinct zones dependent on their relative distance to the liver-metastasis boundary.

**Figure 5:**
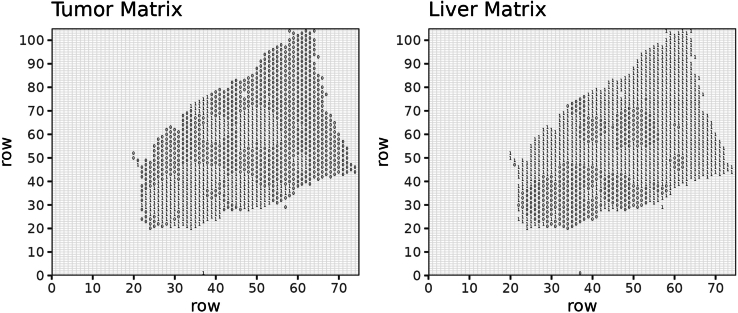
Example for the binary matrices of tumor (left) and liver (right) of sample 4

**Figure 6:**
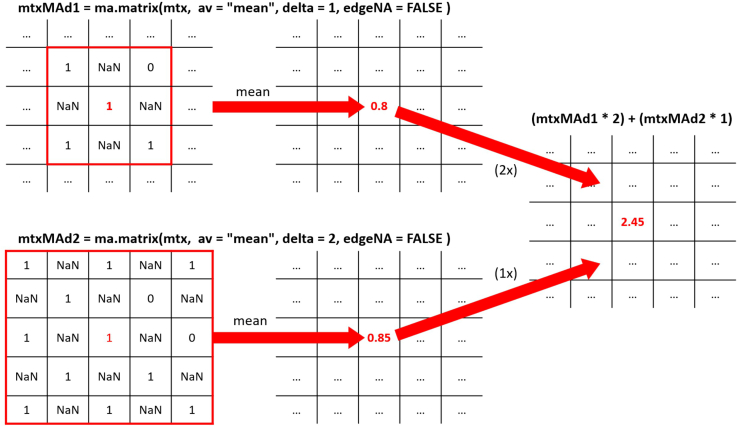
Schematics showing an example of how the matrix is computed when parameters are defined as 'delta' = 1 and 'delta' = 2 The higher the 'delta' is defined, the sharper the definition of the compartmental spot.
  d. Accordingly, create a binary matrix, 'msub1liv' in which all coordinates of spots covering a liver parenchyma area are annotated as 1 and spots covering a metastatic area are annotated as 0.
  e. Create a matrix, 'nameTokeep' in which all coordinates of spots covering tissue are annotated with their 'barcode_ID' and all other positions are annotated as NA. This matrix will be later used in the integrated object.
  f. Compute a moving average on the matrix 'msub1' using the 'ma.matrix' function.
    ***Note:*** For that purpose, the moving average will consider a sliding window encompassing two layers of spots surrounding the spot of interest as well as a moving window encompassing three layers of spots surrounding the spot of interest. The size of the sliding window is defined by the value for 'delta' (here 'delta' = 2 and 'delta' = 3, respectively). Resulting matrixes are multiplied by the factors 2 and 1, respectively, and summed. The value for spots covering liver parenchyma is set to 0 (Figure 6).
  g. Compute a moving average score based on the matrix 'msub1liv' as described above.
    ***Note:*** For that purpose, calculate the moving average considering two layers of spots surrounding the spot of interest, weighted by the factor 2, as well as a moving average considering three layers of spots surrounding the spot of interest, weighted by the factor 0.1, and summarize those two. The value for spots covering metastasis is set to 0.
    Here we show a representative image of the moving average calculated for the individual spots based on the 'msub1' matrix (Figure 7, **left panel)** and the 'msub1liv' matrix (Figure 7, **right panel**) for sample 4.

**Figure 7:**
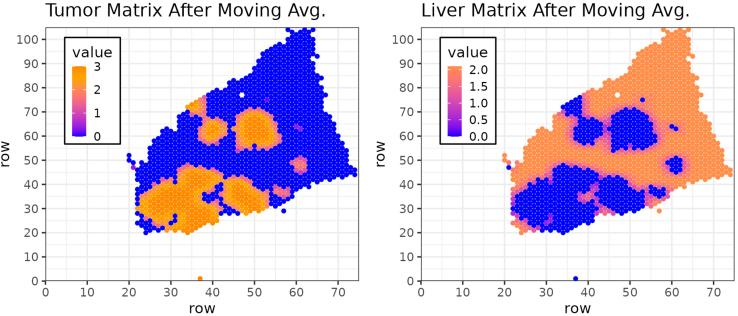
Example of tumor (left) and liver (right) matrices after applying the moving average approach with 'delta' 2 and 3 for sample 4 https://cran.r-project.org/web/packages/reshape2/index.html (H&E staining for the corresponding sample shown in Figure 4) Color represents the value obtained for each position of the matrix.
  h. Assign spots to compartmental zones based on their relative distance from the metastasis/liver parenchyma boundary using the previously calculated moving average matrices.
    ***Note:*** The moving average value for each spot is the sum of two 'ma.matrix' functions which individually result in a value in the range of 0–1. As described previously, these values are multiplied by a defined factor (for 'masubliv' we used 0.1 and 2, and for 'masub1′ we used 1 and 2). Therefore, the resulting value will be in the range of 0–2.1 for the 'masubliv' matrix and 0 to 3 for the 'masub1′ matrix. Here, the score values defining the zones are chosen manually to generate compartmental zones. The optimal choice depends on the users' needs. In this application, the values were chosen to result in compartmental zones with a thickness of approximately one layer of spots, leading to an absolute thickness of about 100 μm per zone in datasets generated using Visium technology (10X). For other technologies such as Visium HD the absolute thickness depends on the size of the individual transcriptomic units as well as the number of layers of transcriptomic units summarized in each zone. If more than 8 zones are required, further score values can be introduced.
  i. Merge the matrices for the metastatic tissue and liver parenchyma tissue and rename the compartmental zones from 'Zone_A' describing the inside of the metastasis to 'Zone_H', the compartmental zone for spots covering distant liver parenchyma, and generate a table containing information about the spot identity (SeuratID) as well as the name of the assigned compartmental zone for each spot.
    ***Note:*** The name of the individual zones can be adjusted to the user's needs.
  j. Merge the zone name of each spot into the Seurat object (integrated) and close the loop.
    #6a:
    for (sample in samples) {
     crc.integrated <- SetIdent(object = crc.integrated, value = "orig.ident")
     sub1 <- subset(crc.integrated, idents = sample)
     sub1 <- SetIdent(object = sub1, value = "integrated_FinalClusters")
     Coord <- merge(crc.integrated@images[[paste0(sample,"img")]]@coordinates, crc.integrated@meta.data[crc.integrated@meta.data$orig.ident == sample, "integrated_FinalClusters", drop = F], by = 0)
     rownames(Coord) <- Coord$Row.names
     Coord$Row.names <- NULL
    #6b:
     Coord$ift <- ifelse(Coord$integrated_FinalClusters == 1 | Coord$integrated_FinalClusters == 6, 1, 0)
    # Figure 4
     p <- ggplot(data = Coord, aes(x = row, y = col, label = ift, color = as.factor(ift))) + theme_bw(base_size = 9) + theme(legend.key.size = unit(.2, "cm"), legend.position = c(.85, .15), legend.background = element_rect(color = "black")) + geom_point(size = .8) + scale_x_continuous(n.breaks = 10, expand = c(0, 1)) + scale_y_continuous(n.breaks = 10, expand = c(0, 1)) + scale_color_brewer(palette = "Set1", name = "Ift)
     ggsave(filename = paste(out_dir, paste("UMAP_", sample,"_IFT.png", sep = ""), sep = "/"), plot = p, width = 8, height = 7, units = "cm", dpi = 300)
    #6c:
     maxrow <- max(Coord$row)
     maxcol <- max(Coord$col)
     msub1 <- matrix(rep(NA, maxrow ∗ maxcol), ncol = maxcol)
     msub1 <- as.data.frame(msub1)
     for (i in c(1:length(Coord$ift))) {
     msub1[Coord$row[i], Coord$col[i]] <- Coord$ift[i]
    }
    #6d:
     msub1liv <- matrix(rep(NA, maxrow ∗ maxcol), ncol = maxcol)
     msub1liv <- as.data.frame(msub1liv)
     for (i in c(1:length(Coord$ift))){
     msub1liv[Coord$row[i], Coord$col[i]] <- (-Coord$ift[i]+1)
    }
    # Figure 5 (left): Tumor Matrix.
     mma <- as.matrix(msub1)
     rownames(mma) <- seq(1, nrow(mma))
     colnames(mma) <- seq(1, ncol(mma))
     mma <- reshape2::melt(mma)
     p <- ggplot(data = mma, aes(x = Var1, y = Var2, label = value)) + theme_bw(base_size = 10) + theme(legend.position = "none", panel.grid.major = element_line(linewidth = .2)) + geom_text(size = 1, family = "mono") + xlab("row") + ylab("row") + ggtitle("Tumor Matrix") + scale_x_continuous(n.breaks = 10, expand = c(0, 1), minor_breaks = seq(1, max(mma$Var1))) + scale_y_continuous(n.breaks = 10, expand = c(0, 1), minor_breaks = seq(1, max(mma$Var2)))
     ggsave(filename = paste(out_dir, paste("Matrix_", sample, "_Tumor.png", sep = ""), sep = "/"),
     plot = p, width = 8, height = 7, units = "cm", dpi = 300)
    # Figure 5 (right): Liver Matrix
     mmaliv <- as.matrix(msub1liv)
     rownames(mmaliv) <- seq(1, nrow(mmaliv))
     colnames(mmaliv) <- seq(1, ncol(mmaliv))
     mmaliv <- reshape2::melt(mmaliv)
     p <- ggplot(data = mmaliv, aes(x = Var1, y = Var2, label = value)) + theme_bw(base_size = 10) + theme(legend.position = "none", panel.grid.major = element_line(linewidth = .2)) + geom_text(size = 1, family = "mono") + xlab("row") + ylab("row") + ggtitle("Liver Matrix") + scale_x_continuous(n.breaks = 10, expand = c(0, 1), minor_breaks = seq(1, max(mma$Var1))) + scale_y_continuous(n.breaks = 10, expand = c(0, 1), minor_breaks = seq(1, max(mma$Var2)))
     ggsave(filename = paste(out_dir, paste("Matrix_", sample, "_Liver.png", sep = ""), sep = "/"),
     plot = p, width = 8, height = 7, units = "cm", dpi = 300)
    #6e:
     namesTokeep <- matrix(rep(NA, maxrow ∗ maxcol), ncol = maxcol)
     namesTokeep <- as.data.frame(namesTokeep)
     for (i in c(1:length(Coord$ift))){
     namesTokeep[Coord$row[i], Coord$col[i]] <- rownames(Coord)[i]
     }
    #6f:
     masub1 <- ma.matrix(as.matrix(msub1), av = "mean", delta = 2, edgeNA = FALSE )
     masub1 <- ma.matrix(as.matrix(msub1), av = "mean", delta = 3, edgeNA = FALSE ) + (masub1∗2)
     indexes1 <- msub1
     masub1 <- masub1 ∗ indexes1
    #6g:
     masub1liv <- ma.matrix(as.matrix(msub1liv), av = "mean", delta = 2, edgeNA = FALSE)
     masub1liv <- ma.matrix(as.matrix(msub1liv), av = "mean", delta = 3, edgeNA = FALSE) ∗ 0.1 + (masub1liv ∗ 2)
     indexes1 <- msub1
     masub1liv <- masub1liv ∗ (-1∗(indexes1-1))
    # Figure 7 (left): Tumor MA Matrix
     mma <- as.matrix(masub1)
     rownames(mma) <- seq(1, nrow(mma))
     colnames(mma) <- seq(1, ncol(mma))
     mma <- reshape2::melt(mma)
     p <- ggplot(data = mma, aes(x = Var1, y = Var2, color = value)) + theme_bw(base_size = 9) + theme(legend.position = c(.15, .75), legend.background = element_rect(color = "black"), legend.key.size = unit(3, "mm")) + geom_point(size = .75) + xlab("row") + ylab("row") + ggtitle("Tumor Matrix After Moving Avg.") + scale_x_continuous(n.breaks = 10, expand = c(0, 1)) + scale_y_continuous(n.breaks = 10, expand = c(0, 1)) + scale_color_gradient2(low = "blue", high = "darkred", mid = "orange", na.value = NA, midpoint = 2.5)
     ggsave(filename = paste(out_dir, paste("Matrix_", sample, "_TumorMA.png", sep = ""), sep = "/"), plot = p, width = 8, height = 7, units = "cm", dpi = 300)
    # Figure 7 (right): Liver MA Matrix
     mmaliv <- as.matrix(masub1liv)
     rownames(mmaliv) <- seq(1, nrow(mmaliv))
     colnames(mmaliv) <- seq(1, ncol(mmaliv))
     mmaliv <- reshape2::melt(mmaliv)
     p <- ggplot(data = mmaliv, aes(x = Var1, y = Var2, color = value)) + theme_bw(base_size = 9) + theme(legend.position = c(.15, .75), legend.background = element_rect(color = "black"), legend.key.size = unit(3, "mm")) + geom_point(size = .75) + xlab("row") + ylab("row") + ggtitle("Liver Matrix After Moving Avg.") + scale_x_continuous(n.breaks = 10, expand = c(0, 1)) + scale_y_continuous(n.breaks = 10, expand = c(0, 1)) + scale_color_gradient2(low = "blue", high = "darkred", mid = "orange", na.value = NA, midpoint = 2.5)
     ggsave(filename = paste(out_dir, paste("Matrix_", sample, "_LiverMA.png", sep = ""), sep = "/"), plot = p, width = 8, height = 7, units = "cm", dpi = 300)
    #6h:
     # Score of tumor
     M1 = 2.96
     M2 = 2.7
     M3 = 2.3
     masub1[masub1 > M1] <- (-4)
     masub1[masub1 <= M1 & masub1 > M2] <- (-3)
     masub1[masub1 <= M2 & masub1 > M3] <- (-2)
     masub1[masub1 <= M3 & masub1 > 0] <- (-1)
     # Score of liver
     L1 = 2.095
     L2 = 1.91
     L3 = 1.7
     masub1liv[masub1liv <= L3 & masub1liv > 0] <- 0
     masub1liv[masub1liv <= L2 & masub1liv > L3] <- 1
     masub1liv[masub1liv <= L1 & masub1liv > L2] <- 2
     masub1liv[masub1liv > L1] <- 3
    #6i:
     finalScores <- masub1 + masub1liv
     finalzones <- finalScores
     finalzones[finalzones == (-4)] <- "Zone_A"
     finalzones[finalzones == (-3)] <- "Zone_B"
     finalzones[finalzones == (-2)] <- "Zone_C"
     finalzones[finalzones == (-1)] <- "Zone_D"
     finalzones[finalzones == 0] <- "Zone_E"
     finalzones[finalzones == 1] <- "Zone_F"
     finalzones[finalzones == 2] <- "Zone_G"
     finalzones[finalzones == 3] <- "Zone_H"
     finalzones[finalzones == "NaN"] <- NA
     FinalTable <- data.frame(SeuratID = as.vector(as.matrix(namesTokeep)), datasub1 = as.vector(as.matrix(finalzones)), drow = rep(1:nrow(masub1), ncol(masub1)), dcol = rep(1:ncol(masub1), nrow(masub1))[order(rep(1:ncol(masub1), nrow(masub1)))])
     FinalTable <- na.omit(FinalTable)
    #6j:
    SeuratPos <- names(crc.integrated@active.ident)
    FinalTable <- FinalTable[order(FinalTable$SeuratID),]
    if(!"Zones" %in% colnames(crc.integrated@meta.data)) {
    crc.integrated@meta.data$Zones <- rep("Zone_A", length(SeuratPos))
    }
    crc.integrated@meta.data$Zones[crc.integrated@meta.data$orig.ident == sample] <- FinalTable$datasub1
    }
7. Export metadata and save the final object.

full_md <- crc.integrated@meta.data

gz_out_md <- gzfile(paste(data_dir, "crc.integrated_metadata.csv.gz", sep = "/"), "w")

write.csv(x = full_md, gz_out_md)

close(gz_out_md)

saveRDS(crc.integrated, file = paste(out_dir, "crc.integrated.rds", sep = "/"))

**Figure 8:**
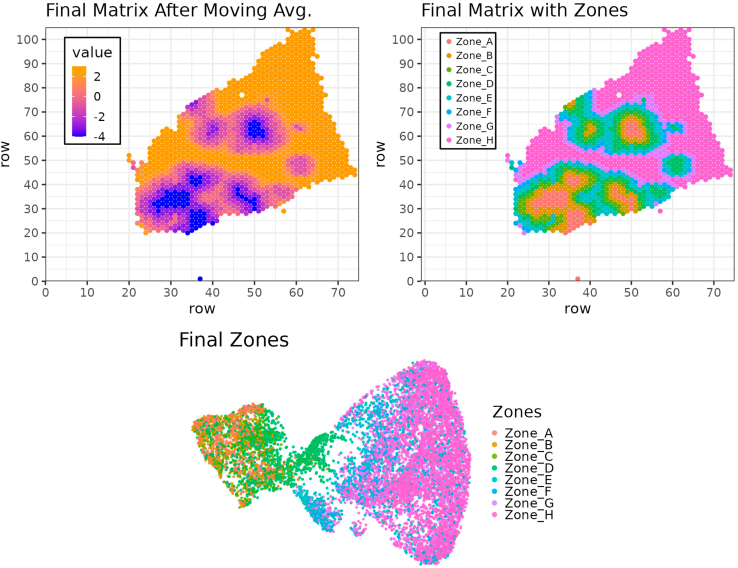
Results of the zonation Results of the moving average strategy obtained by summing the two matrices (e.g., tumor and liver) after applying the moving window average strategy (top left), and the corresponding zones defined on it for sample 4 (top right, H&E staining for the corresponding sample shown in Figure 4), and in the UMAP embeddings of the zones for all samples (bottom).

Here we provide exemplary images for sample 4 representing the combined moving average score calculated for each spatial spot, as well as their annotation into the distinct spatial zones. Furthermore, we show the distribution of the spots belonging to the different zones within the UMAP embedding (Figure 8). Interestingly, there is a trend toward a proximity of spots belonging to the same zones confirming their transcriptomic similarity. However, this similarity is not sufficient to enable the distinction of the zones solely based on the UMAP embedding.

# Figure 8 (top right): Final Matrix with Zones (after Zone assignments)

mmafinal <- as.matrix(finalzones)

rownames(mmafinal) <- seq(1, nrow(mmafinal))

colnames(mmafinal) <- seq(1, ncol(mmafinal))

mmafinal <- reshape2::melt(mmafinal) %>% filter(!is.na(value))

p <- ggplot(data = mmafinal, aes(x = Var1, y = Var2, color = value)) + theme_bw(base_size = 9) + theme(legend.position = c(.15, .75), legend.margin = margin(1,1,1,1), legend.title = element_text(size = 0.1), legend.text = element_text(size = 6), legend.background = element_rect(color = "black"), legend.key.size = unit(3, "mm")) + geom_point(size = .75) + xlab("row") + ylab("row") + ggtitle("Final Matrix with Zones") + scale_x_continuous(n.breaks = 10, limits = c(0, max(mmafinal$Var1)), expand = c(0, 1)) + scale_y_continuous(n.breaks = 10, expand = c(0, 1)) + scale_color_hue(na.value = NA, name = "")

ggsave(filename = paste(out_dir, "Matrix_Sample_FinalZones.png", sep = "/"), plot = p, width = 8, height = 7, units = "cm", dpi = 300)

# Figure 8 (top left): Final Matrix After Moving Avg.

mmafinal <- as.matrix(finalScores)

rownames(mmafinal) <- seq(1, nrow(mmafinal))

colnames(mmafinal) <- seq(1, ncol(mmafinal))

mmafinal <- reshape2::melt(mmafinal)

p <- ggplot(data = mmafinal, aes(x = Var1, y = Var2, color = value)) + theme_bw(base_size = 9) + theme(legend.position = c(.15, .75), legend.background = element_rect(color = "black"), legend.key.size = unit(3, "mm")) + geom_point(size = .75) + xlab("row") + ylab("row") + ggtitle("Final Matrix After Moving Avg.") + scale_x_continuous(n.breaks = 10, expand = c(0, 1)) + scale_y_continuous(n.breaks = 10, expand = c(0, 1)) + scale_color_gradient2(low = "blue", high = "darkred", mid = "orange", na.value = NA, midpoint = 2.5)

ggsave(filename = paste(out_dir, "Matrix_Sample_FinalMA.png", sep = "/"), plot = p, width = 8, height = 7, units = "cm", dpi = 300)

# Figure 8 (bottom): UMAP with Zones (after Zone assignments)

df <- FetchData(object = crc.integrated, c("UMAP_1", "UMAP_2", "Zones"))

df$Zones <- as.factor(df$Zones)

p <- ggplot(data = df, mapping = aes(x = UMAP_1, y = UMAP_2, color = Zones)) + theme_void() + theme(legend.position = "right", legend.key.size = unit(1, "mm")) + ggtitle("Final Zones") + geom_point(size = .25, alpha = .8) + scale_color_hue() + guides(color = guide_legend(override.aes = list(size = 1)))

ggsave(filename = paste(out_dir, "DimPlot_integrated_refined_zones.png", sep = "/"), plot = p, width = 9, height = 6, units = "cm", dpi = 300)

**Figure 9:**
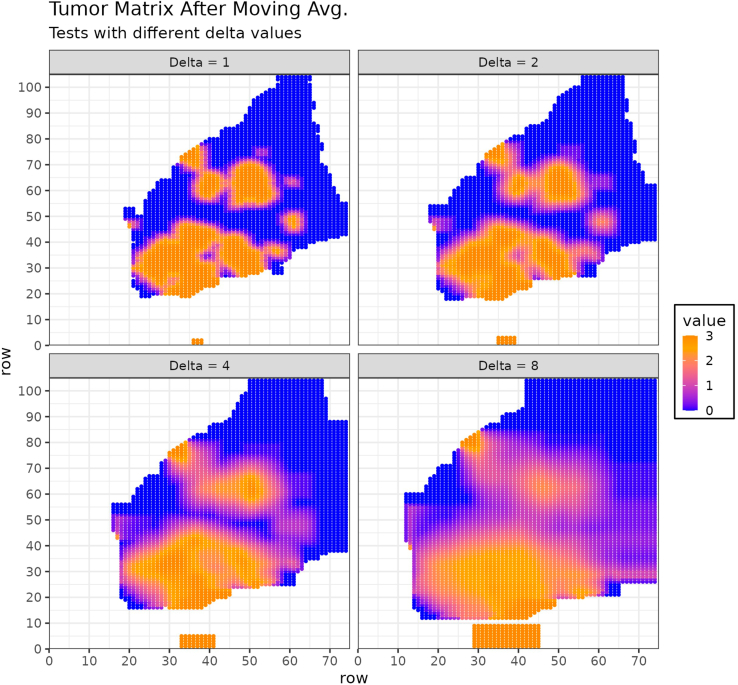
Example of tumor matrices after applying the moving average approach with different 'delta' values (without filtering for the original cells of the matrix) Color represents the value obtained for each position of the matrix.

Here, we provide some examples of the moving average score computed with different values for 'delta' to better visualize the impact of the 'delta' parameter in the moving average strategy used by VisualZoneR (Figure 9). In detail, this parameter corresponds to the determination of the size of the sliding square window, which is (2∗delta+1)x(2∗delta+1). Increasing 'delta' will result in a wider submatrix on which the average is computed. This could help in dealing with sparse dataset or cases in which the distance between spots is higher. Furthermore, a higher 'delta' may be required when using a spatial transcriptomics technology with a smaller spot size leading to a higher resolution such as Visium HD technology. On the other hand, smaller values will reduce the window area, which can help in dealing with smaller populations to characterize.

full.mma <- data.frame()

for (dd in c(1, 2, 4, 8)) {

 masub1 <- ma.matrix(as.matrix(msub1), av = "mean", delta = dd, edgeNA = FALSE )

 masub1 <- ma.matrix(as.matrix(msub1), av = "mean", delta = dd+1, edgeNA = FALSE ) + (masub1∗2)

 mma <- as.matrix(masub1)

 rownames(mma) <- seq(1, nrow(mma))

 colnames(mma) <- seq(1, ncol(mma))

 mma <- reshape2::melt(mma)

 mma$Delta <- paste("Delta =", dd)

 full.mma <- rbind(full.mma, mma)

}

# Figure 9

p <- ggplot(data = full.mma, aes(x = Var1, y = Var2, color = value)) +

theme_bw(base_size = 9) + theme(legend.position = "right", legend.background = element_rect(color = "black"), legend.key.size = unit(3, "mm")) + geom_point(size = .4) + xlab("row") + ylab("row") + ggtitle("Tumor Matrix After Moving Avg.", subtitle = "Tests with different delta values") + scale_x_continuous(n.breaks = 10, expand = c(0, 1)) + scale_y_continuous(n.breaks = 10, expand = c(0, 1)) + scale_color_gradient2(low = "blue", high = "darkred", mid = "orange", na.value = NA, midpoint = 2.5) + facet_wrap(.∼Delta)

ggsave(filename = paste(out_dir, "Matrix_Sample_TumorMA_Tests.png", sep = "/"), plot = p, width = 15, height = 14, units = "cm", dpi = 300)

### Downstream analysis of the data based on the zonation

**Timing: few hours**

Here, we describe examples for downstream analysis of the VisualZoneR pipeline and visualization of the data. The methods described here are focusing on the differential expression of single genes, module score-based analysis or gene set enrichment analysis.

***Note:*** dependent on the user's needs other downstream analysis could be performed.

8. Calculate the differential expression of all genes for each compartmental zone of each treatment cohort.

***Note:*** This code returns differential expression of all genes for all zones. If a more targeted approach is required, the 'FindMarkers' function can be modified according to the user's needs.

crc.integrated@meta.data$Zones_unique <- paste(crc.integrated@meta.data$TreatGroups, crc.integrated@meta.data$Zones, sep = "_")

nClus <- unique(crc.integrated@meta.data$Zones_unique)

DefaultAssay(crc.integrated) <- "SCTintegrated"

crc.integrated <- SetIdent(object = crc.integrated, value = "Zones_unique")

cluster0degAll <- NULL

for (i in nClus) {

 cluster0genes <- FindMarkers(crc.integrated, ident.1 = i, min.pct = 0, only.pos = FALSE, min.cells.group = 1, test.use = "wilcox", return.thresh = 1, logfc.threshold = 1e-11)

 cluster0genes$GeneID <- rownames(cluster0genes)

 cluster0deg <- cluster0genes

 cluster0deg$Cluster <- i

 cluster0degAll <- rbind(cluster0degAll, cluster0deg)

}

write.table(x = cluster0degAll, file = paste(data_dir, "Zones_unique.integrated.txt", sep = "/"), quote = FALSE, row.names = TRUE, sep = "\t")

**Figure 10:**
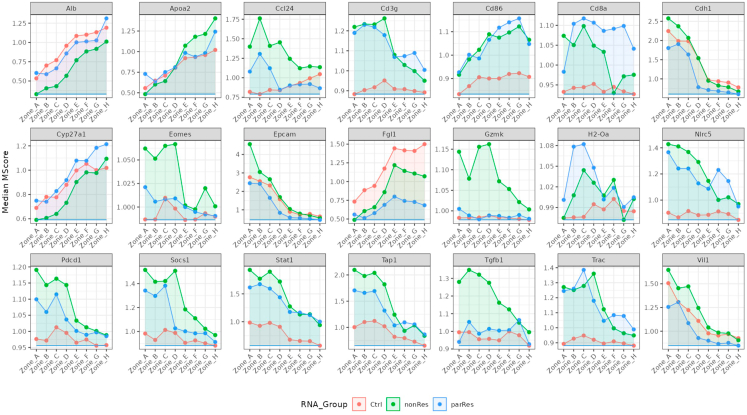
Zonation of selected genes Visualization of expression of the selected genes in the computed zones, colored by group (treatment).

***Note:*** The list of genes can be modified according to the user's needs.

9. Expression of selected genes in the different compartmental zones and treatment cohorts (Figure 10).

genesOI <- c("Epcam", "Cdh1", "Vil1", "Alb", "Apoa2", "Cyp27a1", "Socs1", "Stat1", "Nlrc5", "Fgl1", "Tgfb1", "Ccl24", "Cd86", "H2-Oa", "Tap1", "Cd3g", "Cd8a", "Trac", "Eomes", "Pdcd1", "Gzmk")

full_df_genesOI <- data.frame()

for (geneOI in genesOI) {

 df <- cluster0degAll[cluster0degAll$GeneID==geneOI,]

 full_df_genesOI <- rbind(full_df_genesOI, df)

}

full_df_genesOI$FC <- 2ˆfull_df_genesOI$avg_log2FC

full_df_cor_group_genes <- full_df_genesOI %>% group_by(GeneID, Cluster, FC) %>% summarise(MedianMS = median(FC))

min_gene <- full_df_cor_group_genes %>% group_by(GeneID) %>% summarise(geneMin = min(MedianMS))

full_df_cor_group_genes <- merge(full_df_cor_group_genes, min_gene, by = "GeneID")

full_df_cor_group_genes$Zones <-str_split_fixed(full_df_cor_group_genes$Cluster, "_", 2)[,2]

full_df_cor_group_genes$RNA_Group <-str_split_fixed(full_df_cor_group_genes$Cluster, "_", 2)[,1]

write.xlsx(x = list("MedianGene" = full_df_cor_group_genes[,-5]), file = paste(out_dir, "Full_GenesFC.xlsx", sep = "/"))

# Figure 10.

p <- ggplot(full_df_cor_group_genes, aes(x = Zones, y = MedianMS, color = RNA_Group,fill = RNA_Group, group = RNA_Group, ymin = geneMin, ymax = MedianMS)) + theme_bw() + theme(legend.position = "right", legend.background = element_rect(color = "black"), axis.text.x = element_text(angle = 45, hjust = 1)) + geom_point() + geom_ribbon(position = 'identity', alpha = .1,) + xlab("") + ylab("Median MScore") + facet_wrap(.∼GeneID, ncol = 3, scales = "free")

ggsave(filename = paste(out_dir, "Full_GenesFC.pdf", sep = "/"),

plot = p, width = 10, height = 25)

10. Data representation by plotting module score of selected gene signatures (Figure 11).
  ***Note:*** To run VisualZoneR we used specific gene signatures. Single-cell data can be accessed under:

**Figure 11:**
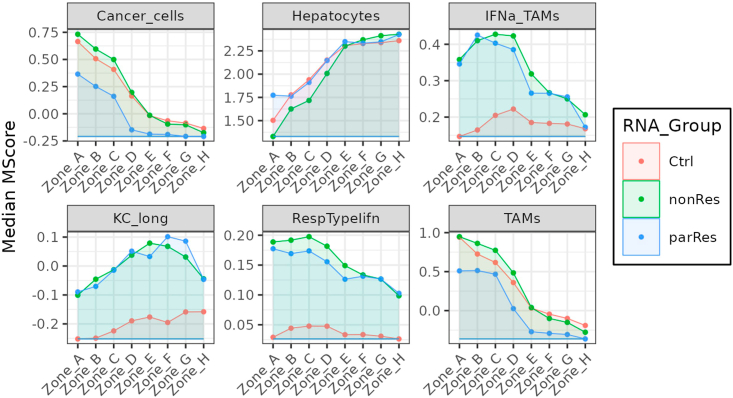
Zonation of selected signatures Visualization of expression of the selected genes signatures in the computed zones, colored by group (treatment).
  https://www.ncbi.nlm.nih.gov/geo/query/acc.cgi?acc=GSE221357. However, other gene sets may be used according to the user’s needs.
  a. Load an object containing all gene sets to be assessed. The file used here can be downloaded from http://www.bioinfotiget.it/gitlab/custom/squadrito_livertumor2022/squadrito_livertumor2022_spatial/-/blob/main/data/miDB_sig3.rds. The file 'miDB_sig3.rds’is placed into the 'data_dir' folder.
    ***Note:*** We used a file containing all gene sets provided at https://www.gsea-msigdb.org/gsea/msigdb/mouse/ as well as some manually added gene sets extracted from Cilenti et al. (https://doi.org/10.1016/j.immuni.2021.05.016) such as the IL-10 macrophage signature. If required, further gene sets can be added to this file.
  b. Calculate and plot of the module score of selected gene signatures.
    #10a:
    miDB_sig2 <- readRDS(paste(data_dir, "miDB_sig3.rds", sep = "/"))
    #10b:
    ifn_sig <- miDB_sig2[["GOBP_RESPONSE_TO_TYPE_I_INTERFERON"]]
    ifn_sig_filt <- ifn_sig[ifn_sig %in% rownames(crc.integrated)]
    signatures <- list("KC_long" = c("Vsig4","Clec4f","Marco","Fcna","Cd5l","C1qa","C1qb","C1qc","Slc40a1","Clec1b","Cd38","Ptgs1","Nr1h3"),
     "TAMs" = c("Lyz2", "Ahnak", "Itgam", "Chil3", "S100a6", "Anxa2","Lyz1", "Mmp7", "Spp1", "Mmp12", "Timp1"),
     "IFNa_TAMs" = c("Ccl2","Chil3","Ly6c2","Arg1","Cxcl10","Slfn4","F10","Lyz2","Msrb1","Hp","Slfn1","Cebpb","Fcgr1","Ifi204","Plac8","Gm21188","Mafb","Tgm2","Ms4a4c","Ifi27l2a"),
     "Cancer_cells" =
    c("Phlda1","Krt18","Krt8","Lars2","Krt19","Epcam","Jun","Cldn7","Lgals4","Cldn4","Cldn3","Krt7","Tcim","Egr1","Sox4","2200002D01Rik","Axin2","Gpx2","Taf1d","Plk2"),
     "Hepatocytes" = c("Fabp1","Apoc1","Apoa2","Mt1","Alb","Serpina1e","Ttr","Gsta3","Serpina1c","Akr1c6","Gstm1","Serpina1a","Serpina1b","Gnmt","Apoc3","Cdo1","Bhmt","Rgn","Ass1","Ttc36"),
     "RespTypeIifn" = ifn_sig_filt)
    for (sig in names(signatures)) {
     crc.integrated <- AddModuleScore(object = crc.integrated, features = list(signatures[[sig]]), name = sig)
    }
    full_df_sig <- data.frame()
    for (sig in names(signatures)) {
     df <- crc.integrated@meta.data[, c(paste0(sig, "1"), "Zones", "TreatGroups", "orig.ident")]
     colnames(df) <- c("Sig", "Zones", "RNA_Group", "Sample")
     df$SigName <- sig
     full_df_sig <- rbind(full_df_sig, df)
    }
    full_df_cor_group <- full_df_sig %>% group_by(SigName, Zones, RNA_Group) %>% summarise(MedianMS = median(Sig))
    min_sig <- full_df_cor_group %>% group_by(SigName) %>% summarise(SigMin = min(MedianMS))
    full_df_cor_group <- merge(full_df_cor_group, min_sig, by = "SigName")
    write.xlsx(x = list("MedianSig" = full_df_cor_group[,-5]), file = paste(out_dir, "Full_SignaturesMedianModScore.xlsx", sep = "/"))
    # Figure 11.
    p <- ggplot(full_df_cor_group, aes(x = Zones, y = MedianMS, color = RNA_Group, fill = RNA_Group, group = RNA_Group, ymin = SigMin, ymax = MedianMS)) + theme_bw() + theme(legend.position = "right", legend.background = element_rect(color = "black"), axis.text.x = element_text(angle = 45, hjust = 1)) + geom_point() + geom_ribbon(position = 'identity', alpha = .1,) + xlab("") + ylab("Median MScore") + facet_wrap(.∼SigName, ncol = 3, scales = "free")
    ggsave(filename = paste(out_dir, "Full_SignaturesMedianModScore.pdf", sep = "/"), plot = p, width = 10, height = 7)
11. Data representation using gene set enrichment analysis visualized in form of a heatmap (Figure 12).
  a. Perform gene set enrichment analysis for each compartmental zone and treatment group using the previously calculated list of differentially expressed genes.
    ***Note:*** The table can be downloaded from the following link:
    http://www.bioinfotiget.it/gitlab/custom/squadrito_livertumor2022/squadrito_livertumor2022_spatial/-/blob/main/data/GSEA_Zones_unique_integrated.res01.txt.
  b. Create a table for each treatment cohort containing the normalized enrichment score (NES) for all analyzed gene sets, sorted from the inside of the tumor towards the tumor-distant parenchyma. The NES value is set to 0 if the adjusted p-value is above 0.05.
  c. Choose manually selected pathways, create a table in which the NES for those pathways for all zones and treatment groups is listed and plot this as a heatmap.
    ***Note:*** The selection of pathways to be displayed can be adjusted according to the user's preferences.
    #11a:
    Clusters <- unique(cluster0degAll[,7])
    TopResultAllCluster <- NULL
    for (i in Clusters) {
    df2 <- cluster0degAll[cluster0degAll[,7] == i, 2]
    names(df2) <- cluster0degAll[cluster0degAll[,7] == i, 6]
     df2 <- df2[order(df2)]
     test1 <- fgsea(pathways = miDB_sig2, stats = df2, minSize = 7, maxSize = 500)
     test1 <- test1[rev(order(NES))]
     TopResult <- as.data.frame(test1[,])[, c(1,2,3,4,5,6,7)]
     TopResult$Cluster <- i
     TopResultAllCluster <- rbind(TopResultAllCluster, TopResult)
    }
    write.table(x = TopResultAllCluster, file = paste(data_dir, "GSEA_Zones_unique_integrated.res01.txt", sep = "/"), quote = FALSE, row.names = TRUE, sep = "\t")
    #11b:
    TopResultAllCluster$Group <- stringr::str_split_fixed(TopResultAllCluster$Cluster, "_", 2)[,1]
    TopResultAllCluster$Zones <- stringr::str_split_fixed(TopResultAllCluster$Cluster, "_", 2)[,2]
    orderC <- unique(TopResultAllCluster$Zones)[order(unique(TopResultAllCluster$Zones))]
    SideColors <- rep(c(rep("darkred",3), rep("red",1), rep("darkgreen",3), rep("green",1)), 3)
    newName <- function(dfCNES,i) {
     rownames(dfCNES) <- dfCNES[, i]
     dfCNES <- dfCNES[, -(i)]
     return(dfCNES)
    }
    for (ix in unique(TopResultAllCluster$Group)) {
     test <- TopResultAllCluster[TopResultAllCluster$Group == ix,]
     test <- test[!is.na(test$padj),]
     test <- test[test$padj < 0.05,]
     AllCluster <- unique(test$Zones)
     orgerGO <- unique(test$pathway)
     dfCNES <- data.frame(pathway = orgerGO[order(orgerGO)])
     for (i in 1:length(AllCluster)){
     dfC1 <- test[test$Zones == AllCluster[i],]
     dfC1 <- data.frame(dfC1[order(dfC1$pathway), c(1, 6)])
     colnames(dfC1) <- c("pathway", AllCluster[i])
     dfCNES <- merge(dfCNES, dfC1, by = 1, all = T)
     }
     dfCNES <- newName(dfCNES, 1)
     dfCNES <- dfCNES[rowSums(is.na(dfCNES)+0) < 8,]
     dfCNES[is.na(dfCNES)] <- 0
     finaldf2 <- as.matrix(dfCNES)
     finaldf2 <- finaldf2[, orderC]
     nameF <- paste(ix, "uniquezones.txt", sep = ".")
     assign(nameF, finaldf2)
     write.table(x = finaldf2, file = paste(out_dir, nameF, sep = "/"), quote = FALSE, row.names = T, sep = "\t")
    }
    #11c:
    Selected.Terms <- c("GOBP_RESPONSE_TO_TYPE_I_INTERFERON","GOBP_RESPONSE_TO_INTERFERON_GAMMA","GOBP_RESPONSE_TO_VIRUS","GOBP_POSITIVE_REGULATION_OF_CYTOKINE_PRODUCTION","GOBP_T_CELL_ACTIVATION","GOBP_ADAPTIVE_IMMUNE_RESPONSE","GOBP_REGULATION_OF_IMMUNE_EFFECTOR_PROCESS","IL10_RO","HALLMARK_ANGIOGENESIS","HALLMARK_P53_PATHWAY","HALLMARK_EPITHELIAL_MESENCHYMAL_TRANSITION","HALLMARK_ADIPOGENESIS","HALLMARK_PEROXISOME","GOBP_SHORT_CHAIN_FATTY_ACID_METABOLIC_PROCESS","GOBP_LIPID_OXIDATION","HALLMARK_BILE_ACID_METABOLISM")
    dfNames <- paste0(unique(TopResultAllCluster$Group),".uniquezones.txt")
    ix3 <- matrix(nrow = length(Selected.Terms), ncol = 0)
    row.names(ix3) <- Selected.Terms
    for (i in dfNames) {
     ix <- read.table(paste(out_dir, i, sep = "/"), header = T, sep = "\t")
     ix <- ix[rownames(ix) %in% Selected.Terms,]
     diffG <- setdiff(Selected.Terms,rownames(ix))
     diffM <- matrix(rep(0, 8∗length(diffG)), ncol = 8)
     rownames(diffM) <- diffG
     colnames(diffM) <- colnames(ix)
     ix2 <- rbind(ix, diffM)
     ix2 <- ix2[Selected.Terms,]
     colnames(ix2) <- paste(sub(".uniquezones.txt", "", i), colnames(ix2), sep = ".")
     ix3 <- cbind(ix3, ix2)
    }
    ix3 <- as.matrix(ix3)
    pdf(paste(out_dir, "Heatmap.uniquezones.pdf", sep = "/"), heigh = 6, width = 8)
    heatmap.2(ix3, margins = c(8, 22), col = viridisLite::viridis(10), cexCol = 1, cexRow = .8, keysize = 1.1, dendrogram = "none", colsep = c(8,16), main = i, scale = "none", Rowv = F, Colv = F, ColSideColors = SideColors)
    legend("topright", legend = c("Inner tumor", "Border tumor", "Peritumor", "Liver"), col = unique(SideColors), lty = 1, lwd = 5, cex = .7)
    dev.off()

**Figure 12:**
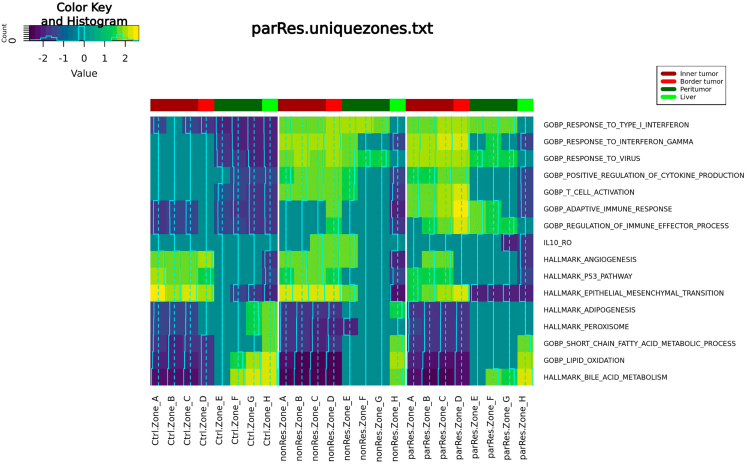
Heatmap showing GSEA analysis on distinct compartmental zones and treatment cohorts

## Expected outcomes

The analysis of spatial transcriptomics data and the incorporation of biological replicates and statistical comparison between distinct treatment cohorts remain challenging. VisualZoneR, the method presented here for the analysis of spatial transcriptomic data from liver metastasis, enables integration of biological replicates and unbiased comparison between different cohorts as well as an assessment of transcriptomic changes between different tumoral and peritumoral areas. The main output of the script is an unbiased classification of spatial spots into distinct compartmental zones within the tumor area and healthy parenchyma dependent on the relative distance from the tumor/parenchyma boundary (Figure 8). However, this script can also be applied to other non-cancer related applications that analyze the transition between two different tissue types. The classification into distinct zones enables several types of downstream analysis including statistical comparison between different treatment cohorts considering biological replicates. Here we describe three potential downstream applications based on (I) the assessment of differential expression of single genes (Figure 10), (II) evaluation of the accumulation of cell types or gene signatures in the different compartmental zones and treatment cohorts based on a module score analysis (Figure 11), and (III) a gene set enrichment analysis addressing treatment related and unrelated upregulation of distinct pathways in different zones (Figure 12). The main expected observations from VisualZoneR applied to the dataset presented in this study are: (I) an enhanced IFNα signaling in IFNα LV treated cohorts, especially within the metastatic and perimetastatic area accompanied with an overall immune activation in these areas; (II) Immune activation in the zones surrounding the metastasis/liver parenchyma boundary especially evident in the Responder cohort while an increased IL10 signaling is observed in the Resistant cohort; (III) accumulation of IFNα-TAMs in the metastatic area as well as of KCs especially in the metastatic and perimetastatic area in IFNα LV treated cohorts.

### Extension to visium HD data

As a proof of concept to fully assess the potential of VisualZoneR, the method has been applied to a dataset generated using Visium HD, a spatial gene expression technology at single cell scale developed by 10X Genomics. In detail, each slide contains two 6.5 × 6.5 mm capture areas with a continuous lawn of oligonucleotides arrayed in ∼11 million 2 × 2 μm barcoded squares without gaps. The data output is provided considering each individual square (bin size of 2 × 2 μm), as well as at multiple bin sizes combining several transcriptomic squares, such as 8 × 8 μm or 16 × 16 μm bins. The dataset employed in the test is the Visium HD Spatial Gene Expression Library, Human Colorectal Cancer (FFPE) from 10X Genomics (available under https://www.10xgenomics.com/datasets?query=&page=1&configure%5BhitsPerPage%5D=50&configure%5BmaxValuesPerFacet%5D=1000&refinementList%5Bproduct.name%5D%5B0%5D=HD%20Spatial%20Gene%20Expression). Analyses were performed using the output at 16 μm resolution (although it was successfully tested at 8 μm resolution as well). Considering that this dataset consists of only one slide, the steps of integrating the data from different slides into one object (steps 4a and b) have been skipped. Based on the structure of the data, we foresee that adjustments of the code for these steps would not be necessary for Visium HD datasets.

The only required adjustment to the previously described procedure involves removing the "empty" spaces in the initial matrices ('msub1′ and 'msub1liv'; steps 6b–6d), thereby avoiding the presence of NA values. To do this, it is possible to take advantage of the cell names coming from the Space Ranger software, which contain the row and column positions of the matrix. For that purpose, the following code could be added between the steps 6b and 6c.

# Coord data frame from step 6b

Coord$row_orig <- Coord$row # save orginal row value

Coord$row <- as.numeric(str_split_fixed(gsub("s_016um_", "", gsub("-1", "", rownames(Coord))), "_", 2)[,2])

Coord$col_orig <- Coord$col # save original col value

Coord$col <- as.numeric(str_split_fixed(gsub("s_016um_", "", gsub("-1", "", rownames(Coord))), "_", 2)[,1])

# continue with step 6c

**Figure 13:**
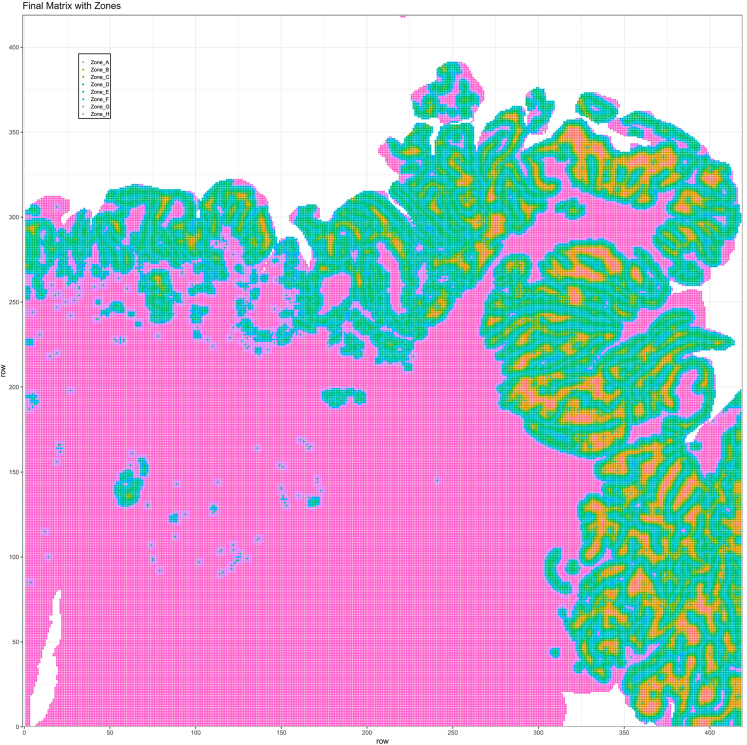
Results of the zonation on Visium HD Result of the VisualZoneR procedure on the Visium HD Human Colorectal Cancer (FFPE) dataset with the corresponding zones defined on it.

To work with a finer resolution of the Visium HD datasets, such as 8 or 2 μm, it is necessary to adjust the cell id prefix in the above instructions from “s_016_um_” to “s_008_um_” or “s_002_um_”, respectively, to correctly extract row and column coordinates. The final zones produced by VisualZoneR on the Human Colorectal Cancer (FFPE) dataset at 16 μm resolution are shown in Figure 13.

This adaptation of VisualZoneR for application to Visium HD datasets enables a broader and a very promising usage of VisualZoneR, especially considering that, at the moment of preparation of this work, Visium HD is a recently developed technology and a very powerful method in the field of spatial transcriptomics. Application of VisualZoneR to datasets generated using other spatial transcriptomics technologies need to be individually adapted and implemented.

## Limitations

In general, this pipeline can be applied to a wide range of spatial transcriptomic analyses where the transition between two tissue types such as metastasis and liver parenchyma is investigated. However, the initial step of the analysis involves the unbiased differentiation of spots covering the two tissue types. This requires that the two analyzed tissue types are clearly distinguishable. Thus, spots covering the different tissues should ideally be either represented in separate clusters in the UMAP representation (Step 5) or express transcriptional features that enable their unbiased identification.

## Troubleshooting

### Problem 1

When data are downloaded from the GEO server, the location and the names of the files may be modified and therefore not fit directions noted in this script.

### Potential solution

• Make sure that the names of the input data match the file names generated in the import directory as described in step 3 of the script and the files are stored at the location defined as 'geo_dir'.

### Problem 2

The gene expression pattern observed in the different clusters in step 5 may not enable a perfect annotation of each cluster as belonging to the metastatic or the liver parenchyma compartment.

### Potential solution

• If the clustering does not distinguish spots covering metastatic or liver parenchyma area (or the tissue types of interest to the user in the dataset), the resolution of the FindClusters command in step 4c can be adjusted.
• If the markers used in step 5 do not allow a clear annotation as metastatic or liver parenchyma for each cluster, additional markers could be included. As mentioned in the manuscript, the list of genes can be modified according to the user’s need to enable distinction of the two tissue types of interest.

### Problem 3

Dependent on the application the user may require a less detailed or more detailed distribution into different zones.

### Potential solution

• The moving average function described in steps 6f (metastatic area) and 6g (liver parenchyma area) is the core of the zonation. If the influence of more distant spots is necessary, the user could increase 'delta' or its multiplication factor. Figure 9 describes the influence of different 'delta' values.
• Furthermore, score values by which spots are clustered into individual zones (6h) can be adjusted to the user’s needs. Of note, the score values chosen here lead to a width of each zone of about 100 μm, which is equal to the distance between spatial transcriptomic spots in datasets generated using Visium technology. Therefore, a more detailed zonation is not feasible here. Other spatial transcriptomic technologies such as Visium HD enable a higher resolution and more detailed zonation. Implementation of more zones e.g., further separating the zones A (inner metastasis) and zone H (tumor distant liver parenchyma) is possible if needed.

### Problem 4

Handling large datasets and images may result in a high consumption of system memory, which might result in an out-of-memory error, thus crashing the task in execution or the entire session in R.

### Potential solution

• Close any other processes or programs, which are running in the system that are not necessary to run code.
• Remove from the R environment objects that are currently non-required using the function 'rm(object_name)'.

### Problem 5

Distinct R versions or R packages may result in minor changes in the results, or even errors that prevent the code from working. Using a different working environment may also influence the clustering observed in step 4c.

### Potential solution

• We don’t believe this might be a real problem. The operator could perform other steps, for example the resolution used for the FindClusters command in step 4c and manual merging of clusters in 4d may be manually adjusted.
• In case the code originates an error message, it might be necessary to use the indicated version of either some of the packages or R.

## Resource availability

### Lead contact

Further information and requests for resources and reagents should be directed to and will be fulfilled by the lead contact, Mario Leonardo Squadrito (squadrito.mario@hsr.it).

### Technical contact

Questions about the technical specifics of performing the protocol should be directed to the technical contact, Stefano Beretta (beretta.stefano1@hsr.it).

### Materials availability

This study did not generate new unique reagents.

### Data and code availability

• Data have been deposited and can be downloaded under https://www.ncbi.nlm.nih.gov/geo/query/acc.cgi?acc=GSE221359.
• The R script generated in this study is available under http://www.bioinfotiget.it/gitlab/custom/squadrito_livertumor2022/squadrito_livertumor2022_spatial/-/blob/main/scripts/VisualZoneR_analysis.R.
